# How COVID-19 News Affect Older Adults’ Mental Health—Evidence of a Positivity Bias

**DOI:** 10.3390/ijerph20053950

**Published:** 2023-02-23

**Authors:** Zoe Ziyi Ng, Grace Li, Suzanne Flynn, W. Quin Yow

**Affiliations:** 1 Raffles Institution, 1 Raffles Institution Lane, Singapore 575954, Singapore; 2 Palo Alto Senior High School, 50 Embarcadero Road, Palo Alto, CA 94301, USA; 3 Department of Linguistics and Philosophy, School of Humanities, Arts, and Social Sciences, Massachusetts Institute of Technology, 77 Massachusetts Avenue, Cambridge, MA 02139, USA; 4 Humanities, Arts and Social Sciences, Singapore University of Technology and Design, 8 Somapah Road, Singapore 487372, Singapore

**Keywords:** COVID-19 news, mental health, older adults, positivity bias, negativity bias

## Abstract

Background: Media affects the trajectory of many individuals’ mental health—with media news, individuals experience negative bias more than positive bias. However, there is also evidence of an age-related positivity effect, with negativity bias generally fading with age. With the rise of COVID-19 cases, older adults (aged 55 years and older) who consume media frequently are at a high risk for declining mental health. To date, there has been no research on the positivity vs. negativity bias of media news on older adults. Here, we investigated whether positivity or negativity bias plays a larger role in affecting how older adults react to COVID-19 news. Methods: Sixty-nine older adults (aged 55–95) answered questions about their weekly media consumption and how closely they followed news relating to COVID-19. They also completed a general health questionnaire. They were then randomly assigned to read either positive or negative COVID-19 news (*n* = 35 and 34, respectively). The adults were asked if the news made them feel happy or fearful, and if they wanted to read more about the news or ignore the news. Results: An analysis revealed that the more often older adults consumed media and the more closely they followed COVID-19 news, the more they felt unhappy and depressed. Importantly, older adults who read positive news reported stronger responses than those who read negative news. Older adults appeared to have a strong positivity bias for COVID-19 news, reporting feeling happy and wanting to read about positive news. In contrast, negative COVID-19 news did not evoke similar levels of response from the older adults. Conclusions: Media consumption of COVID-19 news does negatively impact the mental well-being of older adults, but older adults appear to have a strong positivity bias and a lack of negativity bias for COVID-19 news. These findings suggest that older adults can remain hopeful and positive during periods of public health crises and intense stress, which is essential to sustaining their mental well-being during difficult times.

## 1. Introduction

Past studies suggested that individuals are affected more by negative rather than by positive events or stimuli [1]. For example, selective exposure studies to general news demonstrated that people typically pay more attention to negative news than positive news, leading to a negativity bias in the consumption of media news (e.g., [2,3]). A negativity bias is defined as “a general bias, based on both innate predispositions and experience, in animals and humans, to give greater weight to negative entities” ([4] p. 296). Such a bias is purported to arise due to evolutionary demands, in which attention to negative information helps to initiate a fight-or-flight response that is essential to survival [5,6]. For example, studies have shown that even young infants display a propensity to attend to, learn from, and use negative information far more than positive information [7]. On the other hand, a negativity bias could also arise from socio-cultural learning, in which members of a community feel threatened by uncertainties and conflicts that affect their habitual expectation and attention to negative information [8].

Regardless of the differing theoretical accounts, negativity bias appears to be common and relevant across all human populations and becomes even more prevalent when individuals consume media. For example, negative news tends to disproportionately grab readers’ attention, while good news is treated as no news [9,10]. With the rapid rise of COVID-19 cases in 2020, the media not only provided updates on the pandemic but also fueled hysteria and paranoia in citizens across the world. Devastating updates and statistics dominated the news in all forms of media, especially in traditional media such as television and newspapers. However, consumers of traditional media are now mostly older adults (aged 55 years or older), as younger adults have reduced their reliance on traditional media as sources for information and rely more on the internet and social media platforms [11]. Consequently, traditional media not only served as a source of information for older adults but also became a constant reminder of the devastating effects of the COVID-19 public health crisis for them. Moreover, ref. [12] reported that older adults followed COVID-19 news even more closely than younger adults. Taken together, these studies suggest that older adults might be more susceptible to a negativity bias toward COVID-19 news than younger adults. 

However, there is also evidence of an age-related positivity effect, signifying that the negativity bias in youth fades with age. For example, the socio-emotional selectivity theory proposes that the perception of time influences an individual’s decision-making [13,14,15]. Thus, as an individual ages, they are more inclined to accomplish emotional goals (i.e., enriching social exchange and relationships) versus knowledge-related goals (i.e., learning about the world and environment around them). Instead, positivity bias in media news may exist in older adults because they are more focused on the regulation of their emotions and tend to use cognitive control mechanisms to enhance positive information and diminish negative information. As people age, they report experiencing fewer negative emotions. Studies have also shown a significant decrease in brain activity in response to both positive and negative stimuli, thus eliminating the negativity bias in older adults. For example, ref. [16] examined the negativity bias in 20 younger adults (19–22 years old) and 20 older adults (56–81 years old) during evaluative categorization, focusing on brain activity that occurred approximately 500 milliseconds post visual stimuli. Evaluative categorization is defined as a specific cognitive activity that attributes an appropriate positive or negative value or characteristic to an object based on a mental reference of the object to a certain evaluative category. The results of the study indicated that there is an overall dampening of brain activity associated with the early processing of emotional images, and there was no evidence for the negativity bias in older versus younger adults. Nevertheless, it is not clear whether media news on COVID-19 does, in fact, affect older adults more negatively than positively or vice versa. 

When exposed to the immense stress of negative COVID-19 news, a person’s mental health may be affected. For example, ref. [17] found that a higher level of exposure to disaster news presented on social media sources was associated with a higher level of depression in the Chinese college students who participated in their study. Similarly, the stress that follows from experiencing COVID-19 may be a risk factor that amplifies the detrimental impact of the negativity bias in media news on the mental health of older adults. It has been reported that older adults (aged 55 years and older) were at a high risk for declining mental health during the pandemic, with approximately 40–75% of such community-dwelling older adults reporting suffering from fear, anxiety, and depression [18,19]. A meta-analysis by [20] indicated that the COVID-19 pandemic triggered significantly higher levels of stress, anxiety, and depression across 17 related research studies. This may have become more intense as COVID-19 continued to spread. The authors attributed this increase in the prevalence of stress to the social restrictions that were enacted in Spring 2020 in most countries due to the pandemic. The authors of this study also highlighted the potential for a negative impact on mental health through a cumulative exposure to COVID-related news reported in the media, such that the people who followed COVID-19 news the most also experience the most anxiety [21]. The majority of COVID-19 news is negative; thus, regular exposure to COVID-19 news can lead to an increase level of anxiety [22]. However, the authors did not analyze whether older adults were more vulnerable to the consumption of negative COVID-19 news than younger adults. 

To date, no research has been conducted that focuses on older adults (age 55+) and investigates whether a positivity or negativity bias plays a larger role in affecting the way older adults react to COVID-19 news. Thus, in this study, we seek to investigate how and to what degree media reports concerning positive and negative COVID-19 news affect older adults’ mental health and emotional reactions (measured by the General Health Questionnaire, GHQ-12, and a self-reported survey; see the Methods section for more details). Based on past studies, we designed our study to examine whether 1) the more frequently older adults consume media and follow COVID-19 news, the more they feel unhappy and depressed, and 2) reading positive COVID-19 news leads older adults to feel more happy more than reading negative COVID-19 news would lead them to feel unhappy (positivity bias). 

## 2. Methods

### 2.1. Participants

Sixty-nine older adults (mean age = 69.30 years; median age = 67.50; range = 55–95; 31 males and 38 females; mean age for males = 67.61; mean age for females = 70.68) recruited from the community consented to participate in this study. The study was conducted from January to February 2022. All participants were required to be at least 55 years old, cognitively healthy, and able to read English. The participants were recruited from the Asian community and were concentrated in the Bay Area, California, and Fort Lauderdale, Florida. Participants lived in communities with high Asian populations. Most of the participants had completed a post-secondary/tertiary education (i.e., college or vocational school; 84.1%, *M* = 4.70 out of 5; see Table 1 for details) and were middle-to-high income earners (56.4% reported an annual household income of USD 80,001–120,000 and above). The majority of participants lived with their family members (71%). Participants completed a set of questionnaires (see Appendix A) that asked about their demographics (i.e., age, gender, living status, household income, and highest level of education), media consumption habits, and general health questions (i.e., the General Health Questionnaire, GHQ-12). In addition, participants were randomly assigned to read either a set of negative news (i.e., the Negative condition) or a set of positive news (i.e., the Positive condition) and to complete a set of questions on their responses to the news that they had just read.

Table 1 summarizes the demographic profiles of the participants by condition. For the positive news group, the mean age of 69.50 and age range of 55–95. A majority of participants (72.2%) reported that they lived with family members, with the remaining participants either living alone (22.2%) or living in a senior housing facility (5.6%). The mean household income was 3.31 out of a five-point scale. Similarly, for the negative news group, the mean age was 69.06 and age range was 56–86. A majority of participants (70.6%) reported that they lived with their family members, with the remaining either living alone (26.5%) or living in a senior housing facility (2.9). The mean household income was 2.71. No significant differences were found between the two groups for all the demographic variables listed in Table 1 using independent-sample Mann–Whitney U tests (*p*s > 0.14).

### 2.2. Materials

Background Information Survey. The survey consisted of questions that asked the participants about their age, gender, highest level of education, living status, and socioeconomic status (household income) (see Table 1; all materials can be found in Appendix A).

Media Exposure. This consisted of two questions about the participants’ media consumption habits. For example, the first question in this section asked the participants how many days per week, on average, they consumed media: (1 = 0–1 days; 2 = 2–3 days; 3 = 4–5 days; or 4 = 6–7 days). The second question concerned how closely the participant followed news, specifically about the COVID-19 pandemic, and was measured using a five-point scale (1 = not at all; 5 = very closely).

General Health Questionnaire (GHQ-12; [23,24,25]). The General Health Questionnaire (GHQ-12) is an abridged version, derived from the original 60 item questionnaire, that is commonly used in studies of mental disorders. The 12 item version is often used as a screening measure for current mental health (non-psychotic), has strong psychometric properties, and is extensively used in different settings and different cultures (e.g., [26,27,28,29]). The GHQ asks whether the respondent has recently experienced a particular symptom or emotion (e.g., “Have you been recently able to concentrate on what you’re doing?”, “Have you recently been feeling unhappy and depressed?”). Each item is rated on a four-point scale. There are three ways to score the GHQ-12: the bimodal GHQ scoring method (0-0-1-1) is recommended for use in clinical settings; the C-GHQ scoring method scores the positively phrased items (0-0-1-1) separately from the negatively phrased items (0-1-1-1); and the Likert scoring method (0-1-2-3) is commonly used in research (e.g., [26]). Thus, in our study, we employed the Likert method and scored the items based on (0 = better/healthier than usual, 1 = same as usual, 2 = worse/more than usual, or 3 = much worse/more than usual). This provided a range of total scores from 0 to 36, with higher scores indicating worse mental health conditions. The test authors suggested that there was no need for reverse scoring when using the bimodal GHQ or Likert scoring method.

COVID-19 News. There were a total of four COVID news articles, adapted from news articles (from online news sources including *The New York Times*, *The Straits Times,* and *KCRA 3 News*). These articles were chosen based on two criteria: (1) they were published within the last month prior to the start of the study and (2) they could be clearly classified as positive or negative news. Two of the stories used were positive, and two were negative. The first positive COVID-19 news article detailed how the booster doses were extremely effective in reducing the number of infections and in preventing hospitalization with the Omicron variant. The second positive COVID-19 news article focused on the US administration’s plans to release COVID-19 vaccine doses. The article urged the states to offer the vaccine to all Americans over the age of 55 years. The first negative COVID-19 news article was concerned with how hospitals were strained and overwhelmed by the COVID-19 Omicron surge. The second negative COVID-19 news article was about the number of deaths, especially in older adults, that had occurred from COVID-19 as the pandemic approached the end of its second year.

Response to COVID-19 news questionnaire. The questionnaire administered to all participants after reading the articles inquired about how the participant felt in response to each of the two COVID-19 news articles they had just read. There were a total of four questions per news article: (1) whether the article made them feel happy, (2) if they wanted to read more about the subject discussed in the article, (3) if the news article made them feel fearful, and (4) whether they wanted to forget or ignore the news article. Responses to the questions were scored using a five-point scale (1 = Strongly Disagree; 5 = Strongly Agree). As the third and fourth questions were negatively framed, they were reverse scored (e.g., a score of 5 was converted to 1, 4 was converted to 2, and so on) such that a composite score, i.e., a positive response score, was computed based on the sum of responses for easy interpretation. Multivariate ANOVA analyses revealed no significant effect concerning whether the COVID news questions were framed positively (Q1 and Q2) or negatively (Q3 and Q4) (*ps* > 0.25) (Note: we ran the same multivariate analyses but found no difference in the results if we reverse scored the positively framed questions instead). The maximum positive response score was 40, where higher scores represented stronger positive responses. The Cronbach alpha for this scale was 0.81.

## 3. Procedure

The 69 participants were given URL links to the *Google* form that corresponded to their random assignment. The participants were first asked to provide their consent to participate in the study. They then complete the demographic questionnaire, followed by the General Health Questionnaire (GHQ-12). After the first two sections were completed, depending on whether they were randomly assigned to the positive or negative survey, they were asked to read two positive or two negative COVID-19 news articles (*n* = 35 and 34 respectively). Subsequently, after reading each story, the participants were asked to respond to four questions about how they felt in response to the articles they had just read. 

### Design

This study was conducted as an online survey during the time period of 29 January to 6 February 2022. The participants were invited to take part in the online survey through *Google* Forms. The following covariates were included in this study: gender, age, highest level of education, living status, and economic status. In our multivariate ANOVA and *t*-test analyses, the GHQ score and positive response scores were the dependent variables and the news condition was the independent variable. We defined “positivity bias” as the tendency to process positive news more strongly than negative news. We examined whether participants in the positive news condition rated significantly higher than the neutral rating compared to those in negative news condition (positivity bias). 

## 4. Results

Preliminary analyses using a multivariate ANOVA revealed no significant effects of age, education, living status, and household income on GHQ and positive response scores (*ps* > 0.17); thus, these demographic variables were not included in subsequent analyses. The means and standard deviations of the variables (i.e., the frequency of media consumption, how closely the participants follow COVID-19 news, the GHQ, and the positive response scores) can be found in Appendix A.

First, in order to understand how much of an impact media consumption had on the participants’ mental health, we ran two-tailed correlational analyses on three key variables: how much older adults consumed media, how closely they followed COVID-19 news, and their GHQ-12 items. The analyses revealed that media consumption and following COVID-19 news have no significant relationships with the overall mental well-being of the older adults (GHQ score). Instead, we found that, specifically, the more often older adults consumed media and the more they closely followed COVID-19 news, the more they felt worse (being unhappy and depressed, Q9 and Q11 respectively), *r* = 0.26; *p* = 0.033 and *r* = 0.29; *p* = 0.016, respectively (see Table 2). The linear regression models of media consumption and following COVID-19 news, used for predicting mental health (Q9 and Q11 combined), were significant: *R^2^* > 0.069; *F*s > 5.00; *p*s < 0.029. No other significant relationships were found.

**Figure 1:**
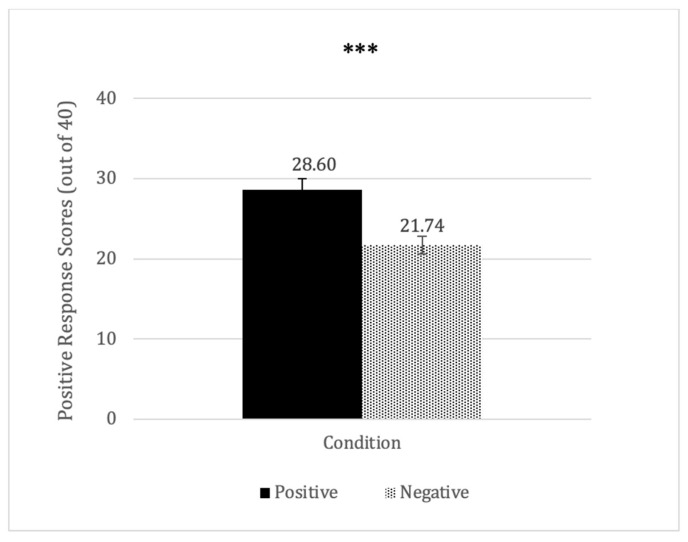
Positive response scores by condition. *** *p* < 0.001.

Next, to determine whether positive and negative COVID-19 news have similar or different effects on older adults’ emotional responses and wellbeing, we ran a multivariate ANOVA with the condition as the independent variable, and the positive response scores and GHQ as the dependent variables. We found there was a significant condition effect on the positive response scores, *F*(1, 67) = 27.16; *η_p_^2^* = 0.39; *p* < 0.001. This effect remained significant even after controlling for media consumption, *F*(1, 65) = 23.28; *η_p_^2^* = 0.26; *p* < 0.001. Older adults who read positive news reported more positive emotions (*M* = 28.6; *SD* = 5.66) than those who read negative news (*M* = 21.74; *SD* = 5.27; see Figure 1). No other significant effects were found. Importantly, to examine whether there were positivity and negativity biases, we first calculated the sum of the delta of a response from a neutral rating (i.e., the absolute difference between the response score and the neutral rating of 3 out of 5) for each participant. We then compared whether participants in the positive news condition had significantly higher delta responses than those in the negative news condition (positivity vs. negativity bias). Independent-samples *t*-tests revealed that participants who read positive news expressed significantly stronger reactions (*M* = 4.60) than participants who read negative news (*M* = 2.26); *t*(67) = 1.77; *p* = 0.040. No other significant results were found. These findings suggest that older adults appeared to have a strong positivity bias for COVID-19 news, reporting feeling happy and wanting to read about positive news. In contrast, negative COVID-19 news did not evoke similar levels of response from the older adults. 

## 5. Discussion

In summary, our findings indicate that the more often older adults consumed media and the more closely they followed COVID-19 news, the more they felt unhappy and depressed. This is largely in line with past studies, suggesting the detrimental impact of general media consumption on mental health. Importantly, we found that positive news evokes significantly stronger reactions than negative news in older adults. In particular, older adults appeared to have a strong positivity bias for COVID-19 news—positive news had a greater impact on older adults than negative news. 

A similar study investigated the age difference in preferences for fear-enhancing and fear-reducing news in a disease outbreak [30]. The results of this study suggested that older adults prefer positive over negative information in a lab setting when compared to young adults (i.e., a positivity effect). In the study, participants responded to an online survey about the COVID-19 pandemic across 13 days during the initial peak of the pandemic in the United States. Both young adults and older adults preferred to read positive news over negative news about the coronavirus, but older adults were more likely than young adults to prefer the positive news article. Importantly, while our study similarly illustrates that older adults have a positivity bias, rather than only having a preference for reading positive news (as per [30]’s study), we found that positive news had a significantly greater impact on older adults than negative news. Our findings suggest that older adults appear to have the ability to remain hopeful and positive during periods of public health crises and intense stress, which is essential to sustaining their mental well-being during difficult times. This is a significant finding, as previous findings reported a positivity effect in which a negativity bias in youth fades with age [14]. Yet, there were no findings from previous studies on the presence of a positivity bias in terms of positive events having a stronger effect than negative events, especially in the context of a public health crisis such as the COVID-19 pandemic.

Our results provide insights into the mindset and outlook on life of older adults. This increased knowledge and understanding of older adults’ positive outlook are important and can be used to our advantage in different areas to possibly reframe how society attempts to improve the mental well-being of older adults during stressful periods by allowing others, e.g., family members and caretakers, to help them more effectively. For example, due to the presence of a positivity bias, it might be beneficial to include more positive news in the media on TV networks, such as *Fox*, *CBS*, and *NBC*, as this could have a greater positive effect on older adults’ mental well-being. Social workers conducting mental health events or campaigns might also make use of this information and encourage older adults to watch and consume both positive and negative news instead of solely watching and reading negative articles. Reading both positive and negative news could overall be beneficial to their mental wellbeing. There is evidence that indicates a positive cognitive bias leads to the maintenance of life satisfaction [31]; this, in turn, helps older adults maintain their mental well-being.

Another possibility is to create special programs that focus on a subset of the news—positive news—for individuals living in separate communities, either orally, online, or in print. Social service agencies can connect with a news studio to present special programs at night or during the day that are specifically targeted to older adults and focus on positive news only. Special news magazines that present only positive news can also be created, and such publications are currently available in schools for students of all ages. These publications could be given a wider circulation. Other ideas include developing and broadcasting special radio shows and podcasts. All these special programs and efforts do not suggest that negative news cannot be shared; rather, it suggests that the news can be structured in such a way that it brings out the positive aspects and is not perceived as negative.

These efforts to reframe the news are critical, as having a positivity bias may be a coping mechanism for older adults in the context of the pandemic. This may be similar to how people’s recollections of the past are often positively biased as a healthy coping mechanism, resulting in people perceiving events in their lives to often be more pleasant than unpleasant [32]. Specifically, ref. [33] examined a sample of UK residents aged 18–55 years on their coping strategies during the COVID-19 pandemic. They found that avoiding negative news about COVID-19 emerged as one of the important coping strategies that served as a protective factor against the negative impact of the pandemic on the residents’ mental and physical health. Such coping strategies are in congruence with other studies, such as [34], who found that avoiding threatening situations significantly reduced health problems and enhanced coping during times of stress and uncertainty. 

One limitation of our study is that we did not include younger adults as a comparison group. Thus, it is not definitive if the positivity bias is, in fact, due to the age-related positivity effect, or if the positivity bias toward COVID-19 news also applies to younger adults. However, in line with the socio-emotional selectivity theory [15], in addition to past studies, which indicated a strong negativity bias among young adults in other domains, we believe that this positivity bias could be an important attribute of older adults. The socio-emotional selectivity theory suggests that as time horizons shrink with age, people become increasingly selective in investing their emotional and intellectual resources in more meaningful goals and activities. This influences cognitive processing, perhaps causing older adults to have a relatively more positive preference for positive over negative information, maximizing their positive emotional experiences and minimizing their emotional risks as they age. Indeed, there are studies that provided evidence that, when compared to younger adults, older adults are more attentive to positive information than negative information (e.g., [14,35], see [36] for a meta-analysis). Nevertheless, it would be beneficial to compare the effects of COVID-19 news on younger adults’ mental health. 

Another possible limitation is that our study was conducted online via Google Forms. We did not print the forms on a hard copy and distribute them to those who do not use internet. Thus, we might have excluded older adults who do not have access to the internet, leading to a skewed representation of the older adults in the USA. However, according to the Pew Research Center [37], 2022, approximately 96% of those aged 50 to 64 and approximately 75% of those aged 65 and older reported being internet users. Our study sample is likely to have captured the majority of the population of older adults. At the same time, our study adopted a between-subjects design, such that participants were randomly assigned to read either positive news or negative news, for two main reasons: (1) to control the independent variables in order to tease out the causal effect of reading positive/negative news and (2) to avoid any order effect, in which reading positive news first might affect how participants read and evaluate subsequent negative news and vice versa. Nevertheless, because participants did not read both types of news articles, they could not act as their own control. Thus, future research should consider reaching out to older adults who do not use internet as well as consider having participants act as their own control due to individual differences.

In this study we focused on traditional media and did not include social media. Recent statistics published by Statista Research Department [38] revealed that, as of April 2022, there were more than five billion internet users worldwide (63.1% of the global population). Of this, 4.7 billion (or 59% of the world’s population) were social media users. Past studies suggested that while social media played an active role in information exchange during COVID-19 times [39,40], it also hastened the spread of misinformation and rumors, which can induce panic, anxiety, and disorientation among the public [41]. Future studies should examine the relationship between social media and its association with mental health during the COVID-19 pandemic. Although early adopters and frequent users of social media are mostly young adults and, as mentioned earlier, older adults are the dominant users of traditional media, social media usage by older adults has increased in recent years as they regularly use it to connect with one another, share information, and engage with news content [42]. This suggests the relevance and significance of social media in the lives of older adults. It remains to be explored whether a similar positivity bias in older adults can also be found in social media usage. 

It is imperative to mention that we had uneven representation of males and females in the two conditions. Although we employed a random assignment for the experimental conditions, the positive news condition had 18 males and 17 females, while the negative news condition had 13 males and 21 females. Similarly, 44.4% of participants in the positive news condition group had an income of USD 160 k+, compared with 17.6% of participants in the negative news condition. However, we did not find any significant differences in male/female participants or the income level between the two conditions. This indicates that, even though the raw numbers are not equal, they are not statistically different. We also ran Chi-square tests using Fisher’s exact test, and no significant difference was found: χ2 = 1.21; *p* = 0.27; Fisher’s exact = 0.336. There were also no statistical differences between groups in terms of gender, income, and age, despite the difference in raw numbers. Even when we added gender and income as factors and age as a covariate in the multivariate ANOVAs, no significant interaction effects were found: *ps* > 0.32. Nevertheless, it is important to point out that there are studies that found evidence of gender differences in the patterns of brain activation of unpleasant stimuli. For example, a meta-analysis of brain imaging studies revealed that women had greater activation in their left amygdala when processing negative stimuli when compared to men [43]. Critically, there are studies that found a stronger negativity bias in women (aged 18–22) than men, in which young women are more attentive to negative news than men [44]. However, it is important to note that (1) despite existing studies alluding to females having a stronger negativity bias compared to adult males, we found a stronger positivity bias than negativity bias even when our sample of older adults consisted of more females than males and (2) these prior studies found gender differences in negativity bias among young adults, but no studies have explored gender differences between positivity and negativity biases toward COVID-19 news among older adults aged 55 and above. 

We also note that our sample was recruited from communities with high Asian populations. This suggests that our results should be interpreted with caution and may not be generalized to populations of other ethnicities. Some studies showed there is no correlation between ethnicity (White, Latinx, and Asian American) and negativity bias [45], yet others found a difference in positivity bias between Caucasians and African Americans, in which Caucasians are more likely to have a positivity bias in than African Americans, and African Americans are more likely to have negativity bias [46]. To our knowledge, there are no studies that examine the influence of gender and ethnicity on positivity and negativity biases in older adults. Future research should consider studying different populations of varying ethnicities and examine whether the strong positivity bias toward media news that we found in our study also exists in populations of other ethnicities.

Finally, we only examined self-reports of emotional outcomes from positive and negative COVID-19 news. The Differential Susceptibility to Media Effects Model (DSMM; [47]) suggests that media usage can influence users’ cognitive, emotional, physiological, and behavioral outcomes. For example, a high media exposure regarding the Ebola pandemic was related to higher level of physiological stress arousal to a prior bomb attack [48]. Future studies could include measures of cognitive and physiological states and health outcomes to better understand the relationships between these variables and the effects of media on them.

## 6. Conclusions 

In conclusion, our findings suggest that older adults have a positivity bias with respect to information and have the ability to remain hopeful during periods of stress. While there are certain limitations to the results, our findings provide novel and valuable insight into the way older adults view information, especially information related to public health crises. This increased understanding will be useful for developing solutions and ideas with respect to maintaining or improving the mental health of older adults, which can ultimately be realized moving forward.

## Figures and Tables

**Table 1:** Demographics of Participants by Condition.

	Positive News Condition	Negative News Condition
Number of Participants	35	34
Number of Males/Females	18/17	13/21
Age (mean in years)	69.50 (*SD* = 11.83)	69.06 (*SD* = 9.18)
Mean highest level of education ^1^ (*SD*)	4.80 (0.53)	4.59 (0.93)
Post-secondary, *n* (%)	30 (86.1%)	28 (82.4%)
High school, *n* (%)	3 (8.3%)	0 (0.0%)
Middle school, *n* (%)	2 (5.6%)	4 (11.8%)
Elementary school, *n* (%)	0 (0.0%)	2 (5.9%)
Preschool or none, *n* (%)	0 (0.0%)	0 (0.0%)
Living Status ^2^		
Significant other, *n* (%)	12 (34.3%)	13 (38.2%)
Family members, *n* (%)	13 (37.1%)	11 (32.4%)
Alone, *n* (%)	8 (22.9%)	9 (26.5%)
Senior housing facility, *n* (%)	2 (5.6%)	1 (2.9%)
Household income ^3^	3.31 (1.73)	2.71 (1.43)
Less than USD 40,000, *n* (%)	10 (28.6%)	8 (23.5%)
USD 40,001–80,000, *n* (%)	2 (5.7%)	10 (29.4%)
USD 80,001–120,000, *n* (%)	5 (14.3.%)	6 (17.6%)
USD 120,001–160,000, *n* (%)	3 (8.6%)	4 (11.8%)
USD 160,000+, *n* (%)	15 (44.4%)	6 (17.6%)

^1^ Highest level of education: 5 = Post-secondary, 4 = High school, 3 = Middle school, 2 = Elementary school, 1 = Preschool or none; ^2^ Living Status: 1 = With their significant other only, 2 = With family members (2+ people), 3 = Alone, 4 = In a senior housing facility; ^3^ Household income: 1 = Less than USD 40,000, 2 = USD 40,001–80,000, 3 = USD 80,001–120,000, 4 = USD 120,001–160,000, 5 = USD 160,000+.

**Table 2:** Pearson correlational analyses between media consumption and well-being.

Pearson (*p*-Value)	Media Consumption ^1^	Following COVID-19 News ^2^	Q9. Feeling Unhappy and Depressed ^3^	Q11. (Not) Feeling Reasonably Happy ^4^	GHQ Score
Media consumption ^1^	-	0.47 *** p < 0.001	0.18 *p* = 0.14	0.29 * p = 0.016	0.16 *p* = 0.19
Following COVID-19 news ^2^	0.47 *** p < 0.001	-	0.26 * p = 0.033	0.17 *p* = 0.18	0.20 *p* = 0.11
Q9. Feeling unhappy and depressed ^3^	0.18 *p* = 0.14	0.26 * p = 0.033	-	0.25 * p = 0.037	0.66 *** p < 0.001
Q11. (Not) Feeling reasonably happy ^4^	0.29 * p = 0.016	0.17 *p* = 0.18	0.25 * p = 0.037	-	0.58 *** p < 0.001
GHQ score	0.16 *p* = 0.19	0.20 *p* = 0.11	0.66 *** p < 0.001	0.58 *** p < 0.001	-

^1^ How many days each week do you consume media? ^2^ How closely do you follow COVID-19 news? ^3^ Have you recently been feeling unhappy and depressed? ^4^ Have you recently been feeling reasonably happy? Note: higher scores in GHQ items indicate worse mental health conditions. The overall GHQ score significantly correlated with all 12 items (*r*s > 0.44; *p*s < 0.001). *** *p* < 0.001; * *p* < 0.05.

## Data Availability

The data presented in this study are available on request from the corresponding author.

## Appendix A

**Table A1:** Survey of the impact of media on the elderly.

A. Personal Information	
i. Gender:	Male Female
ii. Age:	
iii. Highest level of education:	Early childhood education (ex. preschool or daycare) Elementary school Middle school High school Post-secondary/tertiary education (ex. college or vocational school)
iv. Living status:	Living alone With significant other only With family (2+ people) Senior housing facility
v. Economic status (what is your household income):	Less than USD 40,000 USD 40,001–80,000 USD 80,001–120,000 USD 120,001–160,000 USD 160,000+
**B. Media Consumption** i. How many days each week, on average, do you consume media?	0–1 2–3 4–5 6–7
ii. How close do you follow news specifically about the COVID-19 pandemic?	1—Not at all 2 3 4 5—Very closely
**C. General-Health Questionnaire (Select from 1 to 4)** Have you been recently able to concentrate on what you’re doing? Have you recently lost much sleep over worry? Have you recently felt you were playing a useful part in things? Have you recently felt capable of making decisions about things? Have you recently felt constantly under strain? Have you recently felt you couldn’t overcome your difficulties? Have you recently been able to enjoy your normal day-to-day activities? Have you recently been able to face up to your problems? Have you recently been feeling unhappy and depressed? Have you recently been losing confidence in yourself? Have you recently been feeling reasonably happy, all things considered? Have you recently been thinking of yourself as a worthless person?	0—better/ healthier than usual 1—same as usual 2—worse/more than usual 3—much worse/more than usual
**D. COVID-19 News** **(Positive Condition)** i. Read this section of the article “As Omicron Crests, Booster Shots Are Keeping Americans Out of Hospitals”, taken from the New York Times. Booster shots of the Pfizer-BioNTech and Moderna vaccines are not just reducing the number of infections with the highly contagious Omicron variant, they’re also keeping infected Americans out of hospitals, according to data published on Friday by the Centers for Disease Control and Prevention. The extra doses are 90 percent effective at preventing hospitalization with the variant, the agency reported. Booster shots also reduce the likelihood of a visit to an emergency department or urgent care clinic. The data also showed that extra doses are most beneficial against infection and death among Americans ages 50 and older. Overall, the new research indicates that the vaccines are more protective against the Delta variant than against Omicron, which lab studies have found is partially able to sidestep the body’s immune response. “These reports add more evidence to the importance of being up-to-date with COVID vaccinations,” Dr. Rochelle Walensky, director of the C.D.C., said at a White House briefing on Friday.	
This piece of news makes me feel happy.	1—strongly disagree 2 3 4 5—strongly agree
I want to read more about this piece of news.	
This piece of news makes me feel more fearful.	
I want to forget or ignore this piece of news.	
ii. Read this section of the article “US to release COVID-19 doses in push for older Americans to get shots”, taken from the Reuters. The US administration plans to release COVID-19 vaccine doses it has been holding back for second shots and will urge states to offer them to all Americans over age 55, the United States’ top health official said on Tuesday (Jan 12). The move will accelerate distribution of COVID-19 vaccines and jump-start lagging inoculations. The US will expand the availability of shots in community health centers and pharmacies and said the federal government will deploy people to mass vaccination centers. The new strategy would require that Pfizer and partner BioNTech, as well as Moderna—makers of the first two coronavirus vaccines authorized for use in the US—are able to maintain a consistent supply so second shots could still be administered on schedule. Nearly 9 million people in the US were given their first COVID-19 vaccination dose as at Monday, according to the CDC.	
This piece of news makes me feel happy.	1—strongly disagree 2 3 4 5—strongly agree
I want to read more about this piece of news.	
This piece of news makes me feel more fearful.	
I want to forget or ignore this piece of news.	
**(Negative Condition)** i. Read this section of the article “‘We are essentially overwhelmed’: California hospitals strained from COVID-19 omicron surge”, taken from KCRA 3. As omicron cases continue to spike after a busy holiday season, medical providers across Northern California are feeling the strain. “We are essentially overwhelmed,” said Dr. Nicole Braxley, ER medical director at Dignity Health Mercy Hospital. “I get emotional because I think we can do a little better as a community to prevent the spread, just a little bit. The number of COVID-19 positive admissions at Braxley’s hospital tripled in a week. Most patients were not vaccinated. Other health care providers in the area shared similar stories. “We have definitely seen an increase in patient volume and sick patients,” said Siri Nelson, CEO of Marshall Medical Center in Placerville. Nelson said her team is mulling the possibility of turning Marshall into a state surge hospital as case counts continue to climb. The center’s emergency room is already impacted. “In a normal year, we’d be seeing 80 to 85 patients a day,” Nelson explained. “Over the last week, we’ve been seeing over 100 patients a day.” Cindy Rice, vice president of Clinical Nursing Services with Marshal Medical Center, said 75–80% of patients are unvaccinated. “Prevention remains the key. We’re two years in,” Braxley said. “Everyone is tired.”	
This piece of news makes me feel happy.	1—strongly disagree 2 3 4 5—strongly agree
I want to read more about this piece of news.	
This piece of news makes me feel more fearful.	
I want to forget or ignore this piece of news.	
ii. Read this section of the article “As US nears 800,000 COVID-19 deaths, 1 out of every 100 older Americans has perished”, taken from the New York Times. As the coronavirus pandemic approaches the end of a second year, the United States stands on the cusp of surpassing 800,000 deaths from the virus, and no group has suffered more than older Americans. All along, older people have been known to be more vulnerable, but the scale of loss is only now coming into full view. Seventy-five per cent of people who have died of the virus in the US—or about 600,000 of the nearly 800,000 who have perished so far—have been 55 or older. One in 100 older Americans has died from the virus. For people younger than 55, that ratio is closer to one in 1400. In the past two months, the portion of the virus death in older people has risen once again, according to data from the Centres for Disease Control and Prevention (CDC). More than 1200 people in the US are dying from COVID-19 each day, most of them 55 or older.	
This piece of news makes me feel happy.	1—strongly disagree 2 3 4 5—strongly agree
I want to read more about this piece of news.	
This piece of news makes me feel more fearful.	
I want to forget or ignore this piece of news.	

**Table A2:** Means and *SD* of Variables of Interest, Overall and by Condition.

	Overall	Positive News Condition	Negative News Condition
Mean frequency of media consumption ^1^ (*SD*)	3.28 (1.00)	3.51 (0.89)	3.03 (1.06)
Mean of how closely they follow COVID-19 news ^2^ (*SD*)	3.68 (1.16)	3.86 (1.19)	3.50 (1.11)
Mean GHQ Score ^3^ (*SD*)	17.52 (6.03)	17.40 (5.85)	17.65 (6.30)
Mean Positive Response Score ^4^ (*SD*)	25.22 (6.44)	28.60 (5.66)	21.74 (5.27)

^1^ 1 = 0–1 day, 2 = 2–3 days, 3 = 4–5 days, 4 = 6–7 days ^2^ 1 = Not at all to 5 = Very closely ^3^ 0 = less than usual, 1 = no more than usual, 2 = rather more than usual, or 3 = much more than usual ^4^ 1 = Strongly Disagree to 5 = Strongly Agree.
